# Development of a Core Outcome Set for Family and Community Nursing: Protocol for a Delphi Study

**DOI:** 10.2196/51084

**Published:** 2024-03-29

**Authors:** Sara Russo, Rosario Caruso, Gianluca Conte, Arianna Magon, Ida Vangone, Barbara Bascape', Giulia Maga, Malgorzata Pasek, Cristina Arrigoni

**Affiliations:** 1 Department of Biomedicine and Prevention University of Rome Tor Vergata Rome Italy; 2 Health Professions Research and Development Unit IRCCS Policlinico San Donato San Donato Milanese Italy; 3 Department of Biomedical Sciences for Health University of Milan Milan Italy; 4 Nursing Degree Course University of Pavia Istituto Maugeri Istituto di Ricovero e Cura a Carattere Scientifico Pavia Italy; 5 Nursing Degree Course University of Pavia Istituti Clinici di Pavia e Vigevano Pavia Italy; 6 Department of Nursing, Faculty of Health University of Applied Sciences in Tarnów Tarnów Poland; 7 Department of Public Health, Experimental and Forensic Medicine, Section of Hygiene University of Pavia Pavia Italy

**Keywords:** clinical knowledge, core outcomes set, Delphi survey, family and community nurse, health interventions, health promotion, primary care, stakeholder engagement

## Abstract

**Background:**

Family and community nurses (FCNs) play a crucial role in delivering primary care to patients within their homes and communities. A key aspect of their role involves various health interventions, which are influenced by their unique competencies, such as health promotion, advanced clinical knowledge, and strong interpersonal skills. However, it is essential to understand which specific health outcomes these interventions impact to better understand the relationship between FCNs’ skills and the health results.

**Objective:**

This study aims to outline the steps we will take to develop a set of core outcomes. These outcomes will be particularly sensitive to the health interventions carried out by FCNs, providing a clearer picture of their practice’s impact.

**Methods:**

A Delphi survey will be used for this research, conducted from January to December 2024. The process will involve 5 steps and input from 3 stakeholder categories. These stakeholders will help identify a preliminary list of outcomes that will form the basis of our core outcome set (COS).

**Results:**

This guideline will be beneficial for a wide range of stakeholders involved in COS development, including COS developers, trialists, systematic reviewers, journal editors, policy makers, and patient groups. As of January 2024, we have successfully completed the first stage of the study, with the stakeholder group approving the reported outcomes and assigning participant lists for each stakeholder group.

**Conclusions:**

This study will provide a roadmap for identifying the key health outcomes influenced by the interventions of FCNs. The multistakeholder, multiphase approach will ensure a comprehensive and inclusive process. Ultimately, the findings will enhance our understanding of FCNs’ impact on health outcomes, leading to more effective primary care strategies and policies.

**International Registered Report Identifier (IRRID):**

PRR1-10.2196/51084

## Introduction

Family and community nurses (FCNs) provide primary care services to patients within their homes and communities [1]. Their core activities span a broad spectrum of primary services, including health promotion and primary, secondary, and tertiary prevention, focusing on the health and well-being of communities and populations [2]. These specialized professionals are crucial as they deliver accessible and comprehensive primary care services to individuals and communities, particularly in underserved areas and communities with high rates of chronic diseases [3]. Hence, they play a pivotal role in promoting health, preventing disease, and managing chronic conditions by fostering close collaborations with other health care providers to ensure comprehensive patient care. Although various outcomes linked to the activities of FCNs have been described in recent literature [4-8], a formal core outcome set (COS) is yet to be established [9].

Even though FCNs are primarily employed in primary care settings, they have been recognized as key connectors between the various levels of care and health care providers involved in primary, secondary, and tertiary care levels [10]. Primary care is the first level of contact between a patient and the health care system, focusing on illness prevention, health promotion, and the diagnosis and treatment of common conditions [11]. Secondary care involves specialized medical care provided by health care professionals such as specialists, nurses, and technologists, involving more complex medical procedures and treatments in health care facilities [12]. Tertiary care refers to specialized and complex medical and surgical treatments provided by specialized health care providers in specialized centers. In this intricate scenario, FCNs facilitate a smooth transition of care between the various levels of care in a typical patient journey, addressing the challenges of chronic diseases by providing preventive care, health education, and referrals to specialists when necessary [13,14]. This ability to provide comprehensive care and coordinate with other health care providers enables FCNs to ensure that patients receive seamless and effective care across different health care system levels [15].

The curricular competencies of FCNs, as described in recent literature through the ENhANCE project [16], are primarily based on health promotion, high relational competencies, skills, and advanced clinical knowledge. These competencies define the health domain of FCNs as a specific FCN health intervention. According to Caruso and colleagues [16], to frame an integrated view of the complex relationship between curricular competencies and health outcomes, it is necessary to clarify which specific health outcomes are susceptible to the domains described in defining the role of FCNs.

In essence, aligning a specific domain with a health outcome is crucial for 2 main reasons. First, it allows health care providers and policy makers to determine the effectiveness of the FCN role [17]. Second, linking specific domains to specific health outcomes enables the measurement and comparison of the impact of different domains, thereby informing decisions about which domains are most effective for improving health outcomes [18]. Moreover, matching interventions to outcomes helps ensure that resources are used effectively and efficiently [19]. Health care providers and policy makers can prioritize resource allocation and improve health outcomes for the greatest number of people by focusing on domains that impact health outcomes. Lastly, matching domains to outcomes also promotes accountability and transparency in health care [18]. By clearly documenting the impact of domains on health outcomes, health care providers and policy makers can demonstrate their commitment to improving health and provide evidence of their progress toward this goal. However, even though the nature and classification of the health domain provided by FCNs are clearly recognized in the literature (eg, ENhANCE Project - European Curriculum for Family and Community Nurses) [15], the health outcomes that are susceptible to those interventions remain largely undefined and poorly described, and no COS has been defined in relation to the FCNs [9].

A COS is a standardized and agreed-upon set of outcomes that should be measured and reported in all studies of a particular condition or intervention [20]. A COS helps ensure that the most important and relevant outcomes are measured and reported consistently across studies, allowing for meaningful comparison and synthesis of results [20]. Developing a COS involves a systematic and collaborative process involving stakeholders such as patients, health care providers, researchers, and policy makers. The goal is to identify the most important outcomes, starting from a defined framework and developed from the perspective of patients, ensuring that these outcomes are relevant and meaningful to all stakeholders [20,21].

A COS can improve the quality and comparability of research by standardizing the measured and reported outcomes, informing clinical decision-making, and supporting evidence-based health care. It can also help to reduce the risk of selective reporting of outcomes and publication bias, leading to a more comprehensive understanding of the impact of a domain on health outcomes [20,21]. This COS is therefore labeled as the Family and Community Nursing Core Outcomes Set (FCN-COS), using the approach proposed by Moher and colleagues [22]. This protocol will develop FCN-COS sensitive to the health interventions that characterize the practice of FCNs. The objectives of the study are to (1) determine a consensus with a Delphi survey regarding an FCN-COS and (2) develop an FCN-COS sensitive to the health domains that characterize the practice of FCNs involving stakeholders such as patients, health care providers, researchers, and policy makers.

## Methods

### Design

This protocol is designed to incorporate a multimethod and multiphase approach from January to December 2024. It adheres to the recommendations of the “Core Outcome Measures in Effectiveness Trials” (COMET) Handbook (version 1.0) [23] and the Guidance on Conducting and Reporting Delphi Studies (CREDES). The development of the FCN-COS guideline will commence in January 2024 and will be carried out in 5 stages.

- Stage 1: compilation of a preliminary, evidence-based list of reviewed outcomes for consideration in the FCN-COS.
- Stage 2: execution of a web-based Delphi survey to validate the classification and work toward a reporting guideline for FCN-COS.
- Stage 3: conducting a final web-based consensus meeting to identify the definitive outcomes for inclusion in the FCN-COS reporting guideline.
- Stage 4: development of a high-quality reporting guideline and a detailed explanatory document, transforming final FCN outcomes into definitive outcomes and domains.
- Stage 5: Postdevelopment activities, including pilot-testing and dissemination of the results.

The process will involve 3 categories of stakeholders: health care professionals, methodological experts, and service users. These stakeholders will contribute to identifying the preliminary list of proposed outcomes, participate in the Delphi survey rounds, contribute to the consensus meeting, and review the materials produced for disseminating the results.

### Stage 1

#### Preliminary List of Outcomes

The practice of FCNs has significantly contributed to the development of comprehensive health interventions. The preliminary list of proposed outcomes is based on the Classification Clinical Care Classification System (CCC, version 2.5), which provides standard terminology for outcomes and domains, facilitating the documentation of nursing assessments in home care settings. This classification comprises 176 nursing diagnoses, including 60 primary outcomes and 116 diagnostic subcategories. The outcomes included in the preliminary list are defined using existing taxonomies [24], supplemented by a summary of outcomes from a recent systematic review [9] (Multimedia Appendix 1 [5,8,13,25-36]). Additionally, the literature review that will serve as a foundation for developing a preliminary list of outcomes [9] will be updated with specific searches in MEDLINE, CINAHL (EBSCO), Global Health (Ovid), Scopus, and Web of Science. The steering committee will approve the final list of outcomes. The Delphi survey will present the outcomes within each domain to participants in a random order.

#### Preliminary Consensus Meeting

A preliminary consensus meeting has been arranged to define specific methodological aspects of the Delphi survey among the authors of the study protocol. Specifically, an initial web-based consensus meeting was held to define domains. The preliminary outcomes are based on the CCC (version 2.5) taxonomy [24], serving as an evidence-based classification to initiate the preliminary FCN-COS. These preliminary outcomes, developed in the home care setting, have been identified as a crucial starting point [24].

Moreover, a web-based meeting was held in the designing phase of the protocol to discuss strategies for mitigating the risk associated with a low response rate to the Delphi survey. Before starting with the Delphi survey, a brief video will be disseminated among the participants to familiarize them with the project, particularly focusing on the timeline and rounds of discussion.

### Stage 2

#### Delphi Surveys

A web-based Delphi survey will be started to generate consensus regarding the preliminary list of outcomes. We will use the Delphi technique to obtain consensus from the panel of participants through 2 rounds of discussions. Therefore, the first round of discussion will take place from the outcomes identified in the previous stage, inviting participants to add further outcomes not included in the preliminary outcomes. Outcomes identified during the first round will be included in the second round of the Delphi process outcome item. The second round of discussion will take place to provide feedback and identify further outcomes from participants. More precisely, participants will reevaluate their original rating from the first round by including the other participants’ overall rating for each outcome. An outcome will be included in the final FCN-COS with a consensus equal to or greater than 80% in all stakeholder groups rating the outcome as critically important.

#### Participants: Panel Composition

We plan to involve various stakeholder groups, each contributing a balanced mix of skills and competencies. These groups include health care professionals in primary care, health care researchers, nurses actively involved in nursing regulatory authority boards, and service users. The first group will consist of individuals who have experienced primary nursing care within the past 2 years. The second group will focus on research methods to provide robust support for the Delphi survey methodology. Service users will offer feedback based on their experiences in primary care.

#### Recruitment Process

Potential participants will receive personal emails explaining the project and its objectives. Before initiating the survey, we will organize a web-based meeting to help participants better understand the project’s aim. We will then ask them to complete the first round of the Delphi survey within a 2-week time frame. Participation in the survey is optional, and informed consent will be obtained from those who choose to participate. Each participant will be assigned a unique identification number for the 2 rounds of the Delphi survey. We will request the contact authors from each FCN-COS developer stakeholder group to invite their coauthors to provide feedback. This approach ensures that we gather opinions from both clinical and methodological experts in FCN-COS development. At the end of the 2-week period, we will send reminders to participants to complete the survey.

We will collect demographic data, including age, profession, years of experience, educational background, and previous experiences with COS development. Each stakeholder who completes the first round of the Delphi survey will be invited to participate in the second round. We will obtain their name and informed consent to be recognized as a member of the Delphi group in the publication resulting from this research.

#### Delphi Scoring and Software

The reporting guideline outcomes listed will use a 9-point scale, with 1-3 labeled as not important for inclusion in the COS, 4-6 labeled as important but not critical for inclusion in the COS, and 7-9 labeled as critical for inclusion in the COS [37]. Participants will also be allowed to score “maybe” if they are unable to offer an opinion as to whether the reporting guideline outcome is important or not. An electronic survey format called “web-based” will be used for managing the Delphi surveys [23].

#### Data Analysis

Descriptive statistics will be used for each survey round to determine the overall scores for each stakeholder group, considering the 3 categories (not important, important but not critical, and critical) to determine whether outcomes are critical for the FCN-COS. Only responses from participants who will rate at least 50% of the outcomes will be included in the analysis. By comparing the distribution of the mean overall scores from the second Delphi round between participants who attended the consensus meeting and those who did not, selection bias between the Delphi procedure and the consensus meeting (see Stage 3 section) will be evaluated. Data analysis will be performed using SPSS Statistics for Windows (version 28; IBM Corp).

### Stage 3

#### Consensus Meeting

The stakeholder group of 100 people will be included in a web-based interactive consensus meeting. The consensus meeting will be represented through a short study overview as outcomes reported by each stakeholder group. The outcomes where the consensus meeting is reached will be considered first to ratify those results. If a consensus meeting is reached for other outcomes, they will be considered according to the stakeholder groups. An opportunity to discuss each outcome will be given to all participants, defined by an anonymous scoring method, between the ones defined at the consensus meeting. Stakeholders will define the final outcomes based on the review of the responses from Delphi participants. The consensus meeting will be fundamental to identifying the outcomes included in the reporting guideline for FCN-COS development. If 70% of the consensus meeting participants favor the outcome’s inclusion, it will be considered consensus. The best form of communication in the consensus meeting will be determined by comprising methods of feedback (eg, track changes) and ensuring disagreements’ resolution. In the end, a written report after the consensus meeting will be circulated for comment.

#### Delphi Round 1

Each outcome will be presented to reflect the outcomes’ clarity or ambiguity [23]. Every participant will be asked to score each outcome, and after that, they could add any other outcomes they believed to be added to develop FCN-COS. The research group will revise the entire reporting list of outcomes. Each outcome will have a summarized view, including the scored outcome and its distribution. All outcomes will be carried forward to round 2. The response rate of participants as all the participants completed the survey compared to the sent email invitations. The incomplete answers will also be monitored.

#### Delphi Round 2

In the second round, each participant who participated in the first round will be shown the number of respondents and the distribution of scores for each outcome. They will be asked to rescore the outcome of the other Delphi participants and to justify any reason for an eventual change (eg, if a participant changed their score from “not critical” to “critical” in the second round). For each outcome, the number of participants who have scored it and the distribution of scores will be summarized. When the second round is completed, the attrition bias will be considered by comparing 2 groups of the first and second rounds. A comparison will be developed between participants who completed the second round and those who completed only the first one. Changes in participants’ scores will also be examined, and the reasons for changes between the 2 rounds will be summarized.

#### Consensus Definition

A consensus will be defined and obtained before participants take part in the Delphi survey, as previously recommended. Reporting outcomes will only be prioritized if they reach 80% of the support from participants scoring “critical” (ie, score 7-9) [23].

### Stage 4 (Development of Reporting Guideline and Explanatory Document and Procedure)

The aim of the explanatory document is to provide the background, rationale, and justification for the FCN-COS. This document will be developed concurrently with the reporting guidelines.

In stage 4, stakeholders will concentrate on the development of the reporting guideline for studies developing COS and the accompanying explanatory document. Additionally, a draft recommendation will be created to explain the origin of the outcome (whether from stakeholders or the Delphi survey), the level of consensus achieved (through the Delphi survey and consensus meeting), and a brief rationale for inclusion. The expert panel will review the document and provide feedback on the guideline and explanatory document as needed. The FCN-COS guideline will undergo several revisions until the final draft is deemed complete.

### Stage 5 (Pilot-Testing of the FNC-COS)

In the end, in stage 5, we will test the draft completed in the previous stage. Authors will be invited to test the FNC-COS, and the more experienced COS developers will review the guidelines to improve it. Their feedback will be reached through an anonymous survey incorporated into the final COS guideline.

### Ethical Considerations

The University of Pavia Review Board has been consulted and confirmed ethical approval for this study (number 02/int/2023/CdS).

## Results

In this study, a comprehensive FCN-COS will be developed through a rigorous multiphase approach. COS developers, trialists, systematic reviewers, journal editors, policy makers, and patient groups will benefit from the COS development, as it will aim to improve the quality and comparability of future research by standardizing, through 5 distinct stages, the measurement and reporting of outcomes in the field of family and community nursing. As the first stage of the study will be successfully completed, with the stakeholder group expected to approve the reported outcomes and assign participant lists for each stakeholder group, this significant milestone will signify the initial step toward establishing a standardized framework for measuring and reporting outcomes in family and community nursing research, which will inform future clinical decision-making and enhance research quality and comparability in this vital domain. The Delphi survey approach will be a sound method for soliciting input from a wide range of stakeholders, including health care professionals, researchers, and service users. The surveys will aim to establish consensus on the importance of various outcomes. This iterative process will be instrumental in refining the list of outcomes and ensuring that the most critical outcomes are identified. The consensus meeting will mark a critical juncture in the development of the FCN-COS. Bringing together a diverse group of stakeholders for an interactive discussion will ensure that the final outcomes selected truly reflect a collective consensus, as well as the development of the reporting guideline and explanatory document. The involvement of an expert panel in reviewing and revising these documents will ensure their quality and clarity. The pilot-testing stage will represent a valuable step in validating the FCN-COS. Inviting authors and experienced COS developers to test the guideline and provide feedback will be a practical approach to identifying any potential issues or areas for improvement. Their insights will contribute to the refinement and usability of the FCN-COS.

## Discussion

### Overview

To date, no FCN-COS has been developed, and its development could aid in organizing and understanding various interventions within the family and community context [9]. The establishment of FCN-COS will enhance the organization and improvement of FCN interventions, shedding light on the impact of the FCN on health outcomes within a broader family and community context.

This proposed protocol for developing and the future FCN-COS is essential to comprehend how nursing domains are defined by FCN interventions. An example is the “role relationship,” such as parenting skills to ensure child protection. Multidisciplinarity in the health care setting is a priority to ensure continuity of care, involving various health care professionals to better guarantee community health. This can be better illuminated through the FNC-COS, starting from the preliminary version of FCN-COS. The preliminary version of FCN-COS is a crucial starting point. This classification is vital to prioritize the most relevant interventions for FCNs in practice and guide the development of a consensus-based set of core interventions using a Delphi survey approach [24]. This can help ensure that nurses are equipped with the most relevant and evidence-based interventions to provide high-quality care to families and communities.

FCNs provide primary care services to patients in families and communities [1]. Their core activities cover a wide range of primary services, from health promotion to primary, secondary, and tertiary prevention, focusing on the health and well-being of communities and populations [2]. These specialized professionals are important because they provide accessible and comprehensive primary care services to individuals and communities, particularly in underserved areas and communities with high rates of chronic diseases [3]. The FCN is a key role that can bridge the gap between home care settings and hospitals, with autonomy as a self-regulated profession [15]. For this reason, this Delphi process aims to develop a standardized classification of FCN interventions emerging from the unique needs and contexts of FCN practice. In particular, as the FCN’s clinical practice develops in primary, secondary, and tertiary care, defining FCN-COS is a priority that has not yet been developed [9].

The FCN-COS derived from stage 1 is based on the national and international literature [9], and it will need to be adapted for country-specific use in Italy. This will be developed by engaging with a panel of participants in Italy, using a Delphi approach, to define the classification and identification of any outcomes that may be more or less relevant to FCN practice in Italy. In other words, this proposal will be developed as the result of the implementation of the next phases of the protocol to ensure that the FCN is relevant and applicable to the unique needs and contexts of FCN practice in Italy.

### Expected Results From the Delphi Study

Having an FCN-COS is highly relevant to sustaining health promotion and salutogenic interventions in the family and community context [2]. FCN-COS is fundamental to try to fill the gaps about the current unavailability of FCN-COS in the Italian context [9]. This protocol, even if developed in Italy, will be fundamental for the international context. In particular, we focused on the international literature review to define the preliminary outcomes of the FCN-COS [9]. After the preliminary outcomes, stage 1 of the study will follow. A standardized scheme of classification and a precise hierarchical classification of interventions are fundamental to facilitate assessments [21].

Furthermore, the social recognition of the FCN could be strengthened by the definition of a COS shared between health care professionals, even internationally [9,24]. The Delphi survey allows the illumination of experts’ opinions through a series of iterative questionnaires to reach a consensus on defining FCN-COS. The Delphi survey will be conducted ensuring the anonymity of the panel of participants. This is fundamental to reduce the impact of dominant personalities in the debate and decreasing peer pressure [38]. In this sense, every answer will be equally weighted, and the feedback process allows experts to develop a precise idea between one round and another, letting them know the previous ratings of the survey [39].

### Limitations

The methodology used is the main limitation in defining FCN-COS. This approach limited the possibility of elaborating on in-depth views of the panel composition about the FCN-COS. However, a web-based system should help researchers optimize the response rate. Despite this, developing the best possible FCN-COS could be possible through future further validation studies to verify the defined FCN-COS in relation to measurable patient-level outcomes and to the previously developed FCN-COS.

### Conclusion

The Delphi survey is fundamental to developing a COS sensitive to the health interventions that characterize the practice of FCNs. The FCN-COS will be developed as the main turning point, which can determine a greater quality and comparability of research, inform clinical decision-making, and support evidence-based health care. Furthermore, FCN-COS will be fundamental to define FCN as an emerging health care professional in the field of nursing.

## Appendix

Multimedia Appendix 1 Preliminary list of outcomes.

## Abbreviations

CCC: Clinical Care Classification System
COMET: Core Outcome Measures in Effectiveness Trials
COS: core outcome set
CREDES: Guidance on Conducting and Reporting Delphi Studies
FCN: family and community nurse
FCN-COS: Family and Community Nursing Core Outcomes Set

## Data Availability

The data sets generated and analyzed during this study are available from the corresponding author on reasonable request.
