# Correction: Development of a Core Outcome Set for Family and Community Nursing: Protocol for a Delphi Study

**DOI:** 10.2196/60392

**Published:** 2024-06-21

**Authors:** Sara Russo, Rosario Caruso, Gianluca Conte, Arianna Magon, Ida Vangone, Barbara Bascapè, Giulia Maga, Malgorzata Pasek, Cristina Arrigoni

**Affiliations:** 1 Department of Biomedicine and Prevention University of Rome Tor Vergata Rome Italy; 2 Health Professions Research and Development Unit IRCCS Policlinico San Donato San Donato Milanese Italy; 3 Department of Biomedical Sciences for Health University of Milan Milan Italy; 4 Nursing Degree Course University of Pavia Istituto Maugeri Istituto di Ricovero e Cura a Carattere Scientifico Pavia Italy; 5 Nursing Degree Course University of Pavia Istituti Clinici di Pavia e Vigevano Pavia Italy; 6 Department of Nursing, Faculty of Health University of Applied Sciences in Tarnów Tarnów Poland; 7 Department of Public Health, Experimental and Forensic Medicine, Section of Hygiene University of Pavia Pavia Italy

In “Development of a Core Outcome Set for Family and Community Nursing: Protocol for a Delphi Study” (JMIR Res Protoc 2024;13:e51084) the authors noted two errors.

In the originally published manuscript, an error was noted in author affiliation 2:

Health Professions Researchand Development Unit, Istituto di Ricerca e cura a carattere scientifico Policlinico San Donato, San Donato Milanese, Italy

Which has been corrected to the following:

Health Professions Research and Development Unit, IRCCS Policlinico San Donato, San Donato Milanese, Italy

The academic degrees for author Ida Vangone were originally published as "BCS, MSc". This has been corrected to "BSc, MSc".

The correction will appear in the online version of the paper on the JMIR Publications website on June 21, 2024, together with the publication of this correction notice. Because this was made after submission to PubMed, PubMed Central, and other full-text repositories, the corrected article has also been resubmitted to those repositories.

