# Supplementary material for: Development of a Core Outcome Set for Family and Community Nursing: Protocol for a Delphi Study
Source: JMIR Res Protoc. 2024 Mar 29;13:e51084. doi: 10.2196/51084 (PMC11015374; doi:10.2196/51084)
Supplement: Multimedia Appendix 1 [file resprot_v13i1e51084_app1.docx]

**File Supplementary 1.** Preliminary list of outcomes derived from the Clinical Care Classification System for the Core Outcome Set about Family and Community Nursing (FCN-COS) and match with the existing literature

| **Nursing Outcomes** |  | **References** | | | | | | | | | | | | | | |
| --- | --- | --- | --- | --- | --- | --- | --- | --- | --- | --- | --- | --- | --- | --- | --- | --- |
|  |  | **Adamson, 2013** | **Broekema, 2019** | **Dening, 2016** | **Duncan, 2021** | **Falavigna, 2020** | **Holdoway, 2019** | **Husband J, 2008** | **Jin, 2020** | **Murray, 2017** | **Ogston-Tuck, 2018** | **Kelly, 2019** | **Kent, 2011** | **Shafie, 2018** | **Simonetti, 2021** | **Wood-Baker, 2012** |
| Italian wording | English wording |  |  |  |  |  |  |  |  |  |  |  |  |  |  |  |
| Alterazione dell’attività | Activity Alteration |  |  |  |  | x |  |  |  |  |  |  |  |  |  |  |
| Intolleranza all’attività: | Activity Intolerance |  |  |  |  | x |  |  |  |  |  |  |  |  |  |  |
| Rischio di intolleranza all’attività | Activity Intolerance Risk |  |  |  |  | x |  |  |  |  |  |  |  |  |  |  |
| Carenza di attività ricreative | Diversional Activity Deficit |  |  |  |  |  |  |  |  |  |  |  |  |  |  |  |
| Fatigue | Fatigue |  |  |  |  |  |  |  |  |  |  |  |  |  |  |  |
| Compromissione della mobilità | Physical Mobility Impairment |  |  |  | x |  |  |  |  |  |  |  |  |  |  |  |
| Disturbo del modello di sonno | Sleep Pattern Disturbance |  |  |  |  |  |  |  |  |  |  |  |  |  |  |  |

| **Tab 1.** *Cont.*  **Nursing Outcomes** | |  | | | **References** | | | | | | | | | | | | | | | | | | | | | | | | | | | | | | | | | | | | | | | | | | | | | | | | | |  |
| --- | --- | --- | --- | --- | --- | --- | --- | --- | --- | --- | --- | --- | --- | --- | --- | --- | --- | --- | --- | --- | --- | --- | --- | --- | --- | --- | --- | --- | --- | --- | --- | --- | --- | --- | --- | --- | --- | --- | --- | --- | --- | --- | --- | --- | --- | --- | --- | --- | --- | --- | --- | --- | --- | --- | --- |
|  | |  | | | **Adamson, 2013** | | | **Broekema, 2019** | | | **Dening, 2016** | | | **Duncan, 2021** | | | **Falavigna, 2020** | | | **Holdoway, 2019** | | | **Husband J, 2008** | | | **Jin, 2020** | | | **Murray, 2017** | | | **Ogston-Tuck, 2018** | | | **Kelly, 2019** | | | **Kent, 2011** | | | **Shafie, 2018** | | | **Simonetti, 2021** | | | **Wood-Baker, 2012** | | | | | | |  |  |
| Italian wording | | English wording | | |  | | |  | | |  | | |  | | |  | | |  | | |  | | |  | | |  | | |  | | |  | | |  | | |  | | |  | | |  | | | | | | |  |  |
| Privazione di sonno | | | Sleep Deprivation | | |  | | |  | | |  | | |  | | |  | | |  | | |  | | |  | | |  | | |  | | |  | | |  | | |  | | |  | | |  | | | | | | | |
| Alterazione muscoloscheletrica | | | Musculoskeletal Alteration | | |  | | |  | | |  | | |  | | | x | | |  | | |  | | |  | | |  | | |  | | |  | | |  | | |  | | |  | | |  | | | | | | | |
| Alterazione dell’eliminazione intestinale | | | Bowel Elimination Alteration | | |  | | |  | | |  | | |  | | |  | | |  | | |  | | |  | | |  | | |  | | |  | | |  | | |  | | |  | | |  |  |  |  |  |  |  |  |
| Incontinenza intestinale | | | Bowel Incontinence | | |  | | |  | | |  | | |  | | |  | | |  | | |  | | |  | | |  | | |  | | | x | | |  | | |  | | |  | | |  | | | | | | | |
| Diarrea | | | Diarrhea | | |  | | |  | | |  | | |  | | |  | | |  | | |  | | |  | | |  | | |  | | |  | | |  | | |  | | |  | | |  | | | | | | | |
| Intasamento fecale | | | Fecal Impaction | | |  | | |  | | |  | | |  | | |  | | |  | | |  | | |  | | |  | | |  | | |  | | |  | | |  | | |  | | |  | | | | | | | |
| Stipsi percepita | | | Perceived Constipation | | |  | | |  | | |  | | |  | | |  | | |  | | |  | | |  | | |  | | |  | | |  | | |  | | |  | | |  | | |  | | | | | | | |
| Stipsi | | | Constipation | | |  | | |  | | |  | | |  | | |  | | |  | | |  | | |  | | |  | | |  | | |  | | |  | | |  | | |  | | |  | | | | | | | |
| Alterazione gastrointestinale | | | Gastrointestinal Alteration | | |  | | |  | | |  | | |  | | |  | | |  | | |  | | |  | | |  | | |  | | |  | | |  | | |  | | |  | | |  | | | | | | | |
| **Tab 1.** *Cont.*  **Nursing Outcomes** | |  | | | **References** | | | | | | | | | | | | | | | | | | | | | | | | | | | | | | | | | | | | | | | | | | | | | | |  |  |  |  |
|  | |  | | | **Adamson, 2013** | | | **Broekema, 2019** | | | **Dening, 2016** | | | **Duncan, 2021** | | | **Falavigna, 2020** | | | **Holdoway, 2019** | | | **Husband J, 2008** | | | **Jin, 2020** | | | **Murray, 2017** | | | **Ogston-Tuck, 2018** | | | **Kelly, 2019** | | | **Kent, 2011** | | | **Shafie, 2018** | | | **Simonetti, 2021** | | | **Wood-Baker, 2012** | | | |  |  |  |  |  |
| Italian wording | | English wording | | |  | | |  | | |  | | |  | | |  | | |  | | |  | | |  | | |  | | |  | | |  | | |  | | |  | | |  | | |  | | | |  |  |  |  |  |
| Nausea | Nausea | | |  | | |  | | |  | | |  | | |  | | |  | | |  | | |  | | |  | | |  | | |  | | |  | | |  | | |  | | | | | |  |  |  |  |  |  |  |
| Vomito | Vomiting | | |  | | |  | | |  | | |  | | |  | | |  | | |  | | |  | | |  | | |  | | |  | | |  | | |  | | |  | | | | | |  |  |  |  |  |  |  |
| Alterazione della gittata cardiaca | Cardiac Output Alteration | | |  | | |  | | |  | | |  | | |  | | |  | | |  | | |  | | |  | | |  | | |  | | |  | | |  | | |  | | | | | |  |  |  |  |  |  |  |
| Alterazione cardiovascolare | Cardiovascular Alteration | | |  | | |  | | |  | | |  | | |  | | |  | | |  | | |  | | |  | | |  | | |  | | |  | | |  | | |  | | | | | |  |  |  |  |  |  |  |
| Alterazione della pressione sanguigna | Blood Pressure Alteration | | |  | | |  | | |  | | |  | | |  | | |  | | |  | | |  | | |  | | |  | | |  | | |  | | |  | | |  | | |  | | | | | | |  |  |  |
| Rischio di sanguinamento | Bleeding Risk | | |  | | |  | | |  | | |  | | |  | | |  | | |  | | |  | | |  | | |  | | |  | | |  | | |  | | |  | | |  | | | | | | |  |  |  |
| Alterazione della funzione cerebrale | Cerebral Alteration | | |  | | |  | | |  | | |  | | |  | | |  | | |  | | |  | | |  | | |  | | |  | | |  | | |  | | |  | | |  | | | | | | |  |  |  |

| **Tab 1.** *Cont.*  **Nursing Outcomes** | |  | | **References** | | | | | | | | | | | | | | | | | | | | | | | | | | | | | | |
| --- | --- | --- | --- | --- | --- | --- | --- | --- | --- | --- | --- | --- | --- | --- | --- | --- | --- | --- | --- | --- | --- | --- | --- | --- | --- | --- | --- | --- | --- | --- | --- | --- | --- | --- |
|  | |  | | **Adamson, 2013** | | **Broekema, 2019** | | **Dening, 2016** | | **Duncan, 2021** | | **Falavigna, 2020** | | **Holdoway, 2019** | | **Husband J, 2008** | | **Jin, 2020** | | **Murray, 2017** | | **Ogston-Tuck, 2018** | | **Kelly, 2019** | | **Kent, 2011** | | **Shafie, 2018** | | **Simonetti, 2021** | | **Wood-Baker, 2012** | |  |
| Italian wording | | English wording | |  | |  | |  | |  | |  | |  | |  | |  | |  | |  | |  | |  | |  | |  | |  | |  |
| Alterazione della pressione sanguigna | Blood Pressure Alteration | |  | |  | |  | |  | |  | |  | |  | |  | |  | |  | |  | |  | |  | |  | |  | |  |  |
| Rischio di sanguinamento | Bleeding Risk | |  | |  | |  | |  | |  | |  | |  | |  | |  | |  | |  | |  | |  | |  | |  | |  |  |
| Alterazione della funzione cerebrale | Cerebral Alteration | |  | |  | |  | |  | |  | |  | |  | |  | |  | |  | |  | |  | |  | |  | |  | |  |  |
| Confusione mentale | Confusion | |  | |  | |  | |  | |  | |  | |  | |  | |  | |  | |  | |  | |  | |  | |  | |  |  |
| Carenza di conoscenza | Knowledge Deficit | |  | |  | |  | |  | |  | |  | |  | |  | |  | |  | |  | |  | |  | |  | |  | |  |  |
| Carenza di conoscenza sull'esame diagnostico | Knowledge Deficit of Diagnostic Test | |  | |  | |  | |  | |  | |  | |  | |  | |  | |  | |  | |  | |  | |  | |  |  |  |  |
| Carenza di conoscenza sul regime dietetico | Knowledge Deficit of Dietary Regimen | |  | |  | |  | |  | |  | |  | |  | |  | |  | |  | |  | |  | |  | |  | |  |  |  |  |

| **Tab 1.** *Cont.*  **Nursing Outcomes** | | |  | | | | **References** | | | | | | | | | | | | | | | | | | | | | | | | | | | | | | | | | | | | | | | | | | | | | | | | | | | | | |
| --- | --- | --- | --- | --- | --- | --- | --- | --- | --- | --- | --- | --- | --- | --- | --- | --- | --- | --- | --- | --- | --- | --- | --- | --- | --- | --- | --- | --- | --- | --- | --- | --- | --- | --- | --- | --- | --- | --- | --- | --- | --- | --- | --- | --- | --- | --- | --- | --- | --- | --- | --- | --- | --- | --- | --- | --- | --- | --- | --- | --- |
|  | | |  | | | | **Adamson, 2013** | | | | **Broekema, 2019** | | | | **Dening, 2016** | | | | **Duncan, 2021** | | | | **Falavigna, 2020** | | | | **Holdoway, 2019** | | | | **Husband J, 2008** | | | | **Jin, 2020** | | | **Murray, 2017** | | | **Ogston-Tuck, 2018** | | | **Kelly, 2019** | | | | **Kent, 2011** | | | **Shafie, 2018** | | | | **Simonetti, 2021** | | | **Wood-Baker, 2012** | | |
| Italian wording | | | English wording | | | |  | | | |  | | | |  | | | |  | | | |  | | | |  | | | |  | | | |  | | |  | | |  | | |  | | | |  | | |  | | | |  | | |  | | |
| Carenza di conoscenza sul processo patologico | Knowledge Deficit of Disease Process | | | | |  | | | |  | | | |  | | |  | | | |  | | | | |  | | | |  | | | |  | | |  | | | |  | | | |  | | | |  | | |  | | | |  | | |  |  |
| Carenza di conoscenza sul volume di liquidi | Knowledge Deficit of Fluid Volume | | |  | | | | |  | | | |  | | |  | | | |  | | | |  | | | |  | | | |  | | | |  | | | |  | | |  | | |  | | | |  | | |  | | | |  |  |  |  |
| Carenza di conoscenza sul regime farmacologico | Knowledge  Deficit of Medication Regimen | | |  | | | | |  | | | |  | | |  | | | |  | | | |  | | | |  | | | |  | | | |  | | | |  | | |  | | |  | | | |  | | |  | | | |  |  |  |  |
| Carenza di conoscenza sulle precauzioni di sicurezza | | Knowledge Deficit of Safety Precautions | | |  | | |  | | | |  | | |  | | |  | | | |  | | |  | | | |  | | | |  | | |  | | |  | | |  | |  | | |  | | | | | | |  |  |  |  |  |  |  |
| Carenza di conoscenza sul regime terapeutico | | Knowledge Deficit of Therapeutic Regimen | | |  | | |  | | | |  | | |  | | |  | | | |  | | |  | | | |  | | | |  | | |  | | |  | | |  | |  | | |  | | | | | | |  | | | | | |  |

| **Tab 1.** *Cont.*  **Nursing Outcomes** | |  | | **References** | | | | | | | | | | | | | | | | | | | | | | | | | | | | | | | | | | | | | | | | | | | | | | |
| --- | --- | --- | --- | --- | --- | --- | --- | --- | --- | --- | --- | --- | --- | --- | --- | --- | --- | --- | --- | --- | --- | --- | --- | --- | --- | --- | --- | --- | --- | --- | --- | --- | --- | --- | --- | --- | --- | --- | --- | --- | --- | --- | --- | --- | --- | --- | --- | --- | --- | --- |
|  | |  | | **Adamson, 2013** | | | **Broekema, 2019** | | | **Dening, 2016** | | | **Duncan, 2021** | | | **Falavigna, 2020** | | | **Holdoway, 2019** | | | **Husband J, 2008** | | | **Jin, 2020** | | | **Murray, 2017** | | | **Ogston-Tuck, 2018** | | | **Kelly, 2019** | | | **Kent, 2011** | | | **Shafie, 2018** | | | **Simonetti, 2021** | | | **Wood-Baker, 2012** | | | |  |
| Italian wording | | English wording | |  | | |  | | |  | | |  | | |  | | |  | | |  | | |  | | |  | | |  | | |  | | |  | | |  | | |  | | |  | | | |  |
| Carenza di conoscenza sul regime terapeutico | Knowledge Deficit of Therapeutic Regimen | | | |  | | |  | | |  | | |  | | |  | | |  | | |  | | |  | | |  | | |  | | |  | | |  | | |  | | |  | | |  | | | |
| Alterazione nei processi di pensiero | | | Thought Processes Alteration | | |  | | |  | | |  | | |  | | |  | | |  | | |  | | |  | | |  | | |  | | |  | | |  | | |  | | |  | | | |  |  |
| Compromissione della memoria | | | Memory Impairment | | |  | | |  | | |  | | |  | | |  | | |  | | |  | | |  | | |  | | |  | | |  | | |  | | |  | | |  | | |  | | |
| Processo di fine vita | Dying Process | | | | x | | |  | | |  | | |  | | |  | | |  | | | x | | |  | | |  | | |  | | |  | | |  | | |  | | |  | | |  | | | |
| Compromissione del coping familiare | Family Coping Impairment | | | |  | | |  | | |  | | |  | | |  | | |  | | |  | | |  | | |  | | |  | | |  | | |  | | |  | | |  | | |  | | | |

| **Tab 1.** *Cont.*  **Nursing Outcomes** | |  | | **References** | | | | | | | | | | | | | | | | | | | | | | | | | | | | | |  |  |  |
| --- | --- | --- | --- | --- | --- | --- | --- | --- | --- | --- | --- | --- | --- | --- | --- | --- | --- | --- | --- | --- | --- | --- | --- | --- | --- | --- | --- | --- | --- | --- | --- | --- | --- | --- | --- | --- |
|  | |  | | **Adamson, 2013** | | **Broekema, 2019** | | **Dening, 2016** | | **Duncan, 2021** | | **Falavigna, 2020** | | **Holdoway, 2019** | | **Husband J, 2008** | | **Jin, 2020** | | **Murray, 2017** | | **Ogston-Tuck, 2018** | | **Kelly, 2019** | | **Kent, 2011** | | **Shafie, 2018** | | **Simonetti, 2021** | | **Wood-Baker, 2012** | | | |  |
| Italian wording | | English wording | |  | |  | |  | |  | |  | |  | |  | |  | |  | |  | |  | |  | |  | |  | |  | | | |  |
| Coping familiare disfunzionale | Disabled Family Coping | |  | |  | |  | |  | |  | |  | |  | |  | |  | |  | |  | |  | |  | |  | |  | | | |  |  |
| Compromissione del coping individuale | Individual Coping Impairment | |  | |  | |  | |  | |  | |  | |  | |  | |  | |  | |  | |  | |  | |  | | | |  |  |  |  |
| Compromissione della capacità di adattamento | Adjustment Impairment | |  | |  | |  | |  | |  | |  | |  | |  | |  | |  | |  | |  | |  | |  | | | |  | | | |
| Conflitto decisionale | Decisional Conflict | |  | |  | |  | |  | |  | |  | |  | |  | |  | |  | |  | |  | |  | |  | | | |  | | | |
| Coping difensivo | Defensive Coping | |  | |  | |  | |  | |  | |  | |  | |  | |  | |  | |  | |  | |  | |  | | | |  | | | |
| Negazione | Denial | |  | |  | |  | |  | |  | |  | |  | |  | |  | |  | |  | |  | |  | |  | | | |  | | | |

| **Tab 1.** *Cont.*  **Nursing Outcomes** | | |  | | | **References** | | | | | | | | | | | | | | | | | | | | | | | | | | | | | | | | | | | | | | | | | | |  |  |
| --- | --- | --- | --- | --- | --- | --- | --- | --- | --- | --- | --- | --- | --- | --- | --- | --- | --- | --- | --- | --- | --- | --- | --- | --- | --- | --- | --- | --- | --- | --- | --- | --- | --- | --- | --- | --- | --- | --- | --- | --- | --- | --- | --- | --- | --- | --- | --- | --- | --- | --- |
|  | | |  | | | **Adamson, 2013** | | **Broekema, 2019** | | **Dening, 2016** | | | **Duncan, 2021** | | | **Falavigna, 2020** | | | **Holdoway, 2019** | | | **Husband J, 2008** | | | **Jin, 2020** | | | **Murray, 2017** | | | **Ogston-Tuck, 2018** | | | **Kelly, 2019** | | | **Kent, 2011** | | | **Shafie, 2018** | | | **Simonetti, 2021** | | | **Wood-Baker, 2012** | | |  |  |
| Italian wording | | | English wording | | |  | |  | |  | | |  | | |  | | |  | | |  | | |  | | |  | | |  | | |  | | |  | | |  | | |  | | |  | | |  |  |
| Risposta post-traumatica | | Post-Trauma Response | | |  | | | | | | |  | | |  | | |  | | |  | | |  | | |  | | |  | | |  | | |  | | |  | | |  | | |  | | |  | |  |
| Sindrome traumatica da stupro | | Rape Trauma Syndrome | | |  | | | | | | |  | | |  | | |  | | |  | | |  | | |  | | |  | | |  | | |  | | |  | | |  | | |  | | |  | |  |
| Alterazione dello stato spirituale | Spiritual State Alteration | | |  | | |  | |  | |  | | |  | | |  | | |  | | |  | | |  | | |  | | |  | | |  | | |  | | |  | | |  | | | | | | |
| Sofferenza spirituale | Spiritual Distress | | |  | | |  | |  | |  | | |  | | |  | | |  | | |  | | |  | | |  | | |  | | |  | | |  | | |  | | |  | | | | | | |
| Compromissione del coping della comunità | Community Coping Impairment | | |  | | |  | |  | |  | | |  | | |  | | |  | | |  | | |  | | |  | | |  | | |  | | |  | | |  | | | | | |  |  |  |  |
| Lutto | Grieving | | |  | | |  | |  | |  | | |  | | |  | | |  | | |  | | |  | | |  | | |  | | |  | | |  | | |  | | |  | | | | | | |

| **Tab 1.** *Cont.*  **Nursing Outcomes** |  | | **References** | | | | | | | | | | | | | | | | | | | | | | | | | | |  |  |  |  |  |
| --- | --- | --- | --- | --- | --- | --- | --- | --- | --- | --- | --- | --- | --- | --- | --- | --- | --- | --- | --- | --- | --- | --- | --- | --- | --- | --- | --- | --- | --- | --- | --- | --- | --- | --- |
|  |  | | **Adamson, 2013** | | **Broekema, 2019** | | **Dening, 2016** | | **Duncan, 2021** | | **Falavigna, 2020** | | **Holdoway, 2019** | | **Husband J, 2008** | | **Jin, 2020** | | **Murray, 2017** | | **Ogston-Tuck, 2018** | | **Kelly, 2019** | | **Kent, 2011** | | **Shafie, 2018** | **Simonetti, 2021** | | | **Wood-Baker, 2012** | |  |  |
| Italian wording | English wording | |  | |  | |  | |  | |  | |  | |  | |  | |  | |  | |  | |  | |  |  | | |  | |  |  |
| Lutto anticipatorio | | Anticipatory Grieving | |  | |  | |  | |  | |  | |  | |  | |  | |  | |  | |  | |  | | |  | | |  | |  |
| Lutto disfunzionale | | Dysfunctional Grieving | |  | |  | |  | |  | |  | |  | |  | |  | |  | |  | |  | |  | | |  | | |  | |  |
| Alterazione nel volume di liquidi | | Fluid Volume Alteration | |  | |  | |  | |  | |  | |  | |  | |  | |  | |  | |  | |  | | |  | | |  | |  |
| Carenza nel volume di liquidi | | Fluid Volume Deficit | |  | |  | |  | |  | |  | |  | |  | |  | |  | |  | |  | |  | | |  | | |  | |  |
| Rischio di carenza nel volume di liquidi | | Fluid Volume Deficit Risk | |  | |  | |  | |  | |  | |  | |  | |  | |  | |  | |  | |  | | |  | | |  | |  |

| **Tab 1.** *Cont.*  **Nursing Outcomes** |  | | **References** | | | | | | | | | | | | | |  |  |  |
| --- | --- | --- | --- | --- | --- | --- | --- | --- | --- | --- | --- | --- | --- | --- | --- | --- | --- | --- | --- |
|  |  | | **Adamson, 2013** | **Broekema, 2019** | **Dening, 2016** | **Duncan, 2021** | **Falavigna, 2020** | **Holdoway, 2019** | **Husband J, 2008** | **Jin, 2020** | **Murray, 2017** | **Ogston-Tuck, 2018** | **Kelly, 2019** | **Kent, 2011** | **Shafie, 2018** | **Simonetti, 2021** | | **Wood-Baker, 2012** |  |
| Italian wording | English wording | |  |  |  |  |  |  |  |  |  |  |  |  |  |  | |  |  |
| Rischio di carenza nel volume di liquidi | | Fluid Volume Deficit Risk |  |  |  |  |  |  |  |  |  |  |  |  |  |  | |  |  |
| Eccesso nel volume di liquidi | | Fluid Volume Excess |  |  |  |  |  |  |  |  |  |  |  |  |  |  | |  |  |
| Rischio di eccesso nel volume di liquidi | | Fluid Volume Excess Risk |  |  |  |  |  |  |  |  |  |  |  |  |  |  | |  |  |
| Squilibrio elettrolitico | | Electrolyte Imbalance |  |  |  |  |  |  |  |  |  |  |  |  |  |  | |  |  |
| Alterazione della capacità di mantenere la salute | | Health Maintenance Alteration |  |  |  |  |  |  |  | x |  |  |  |  |  |  | | | |
| Ritardo nella crescita | | Failure to Thrive |  |  |  |  |  |  |  |  |  |  |  |  |  |  | |  |  |
| Alterazione del comportamento di ricerca della salute | | Health Seeking Behavior Alteration |  |  |  |  |  |  |  |  |  |  |  |  |  |  | | | |

| **Tab 1.** *Cont.*  **Nursing Outcomes** | |  | | | **References** | | | | | | | | | | | | | | | | | | | | | | | | | |  |  |  |  |  |  |
| --- | --- | --- | --- | --- | --- | --- | --- | --- | --- | --- | --- | --- | --- | --- | --- | --- | --- | --- | --- | --- | --- | --- | --- | --- | --- | --- | --- | --- | --- | --- | --- | --- | --- | --- | --- | --- |
|  | |  | | | **Adamson, 2013** | **Broekema, 2019** | **Dening, 2016** | | **Duncan, 2021** | | **Falavigna, 2020** | | **Holdoway, 2019** | | **Husband J, 2008** | | **Jin, 2020** | | **Murray, 2017** | | **Ogston-Tuck, 2018** | | **Kelly, 2019** | | **Kent, 2011** | | **Shafie, 2018** | | **Simonetti, 2021** | | | | **Wood-Baker, 2012** | |  |  |
| Italian wording | | English wording | | |  |  |  | |  | |  | |  | |  | |  | |  | |  | |  | |  | |  | |  | | | |  | |  |  |
| Alterazione della capacità di mantenere la salute | | | Health Maintenance Alteration | |  |  |  | |  | |  | |  | |  | | x | |  | |  | |  | |  | |  | |  | | | | | | | |
| Ritardo nella crescita | | | Failure to Thrive | |  |  |  | |  | |  | |  | |  | |  | |  | |  | |  | |  | |  | |  | | | |  | |  |  |
| Alterazione del comportamento di ricerca della salute | | | Health Seeking Behavior Alteration | |  |  |  | |  | |  | |  | |  | |  | |  | |  | |  | |  | |  | |  | | | | | | | |
| Alterazione della capacità di gestire la casa | Home Maintenance Alteration | | |  | | | |  |  |  | |  | |  | |  | |  | |  | |  | |  | |  | |  | |  | |  |  |  |  |  |
| Non aderenza | Noncompliance | | |  | | | |  |  |  | |  | |  | |  | |  | |  | |  | |  | |  | |  | | x | | | |  | |  |
| Alterazione della capacità di gestire la casa | Home Maintenance Alteration | | |  | | | |  |  |  | |  | |  | |  | |  | |  | |  | |  | |  | |  | |  | |  |  |  |  |  |

| **Tab 1.** *Cont.*  **Nursing Outcomes** |  | | | **References** | | | | | | | | | | | | | | | | | | | | | | | | | | | | | | | | | | | | | | | | | | | | |  |
| --- | --- | --- | --- | --- | --- | --- | --- | --- | --- | --- | --- | --- | --- | --- | --- | --- | --- | --- | --- | --- | --- | --- | --- | --- | --- | --- | --- | --- | --- | --- | --- | --- | --- | --- | --- | --- | --- | --- | --- | --- | --- | --- | --- | --- | --- | --- | --- | --- | --- |
|  |  | | | **Adamson, 2013** | | **Broekema, 2019** | | **Dening, 2016** | | | **Duncan, 2021** | | **Falavigna, 2020** | | | **Holdoway, 2019** | | | **Husband J, 2008** | | | **Jin, 2020** | | **Murray, 2017** | | | **Ogston-Tuck, 2018** | | | **Kelly, 2019** | | | **Kent, 2011** | | | **Shafie, 2018** | | | **Simonetti, 2021** | | | **Wood-Baker, 2012** | | | | | |  |  |
| Italian wording | English wording | | |  | |  | |  | | |  | |  | | |  | | |  | | |  | |  | | |  | | |  | | |  | | |  | | |  | | |  | | | | | |  |  |
| Alterazione della capacità di gestire la casa | | Home Maintenance Alteration | |  | | | | | |  | |  | |  | | |  | | |  | | |  | | |  | | |  | | |  | | |  | | |  | | |  | | |  | |  |  |  |  |
| Non aderenza | | Noncompliance | |  | | | | | |  | |  | |  | | |  | | |  | | |  | | |  | | |  | | |  | | |  | | |  | | |  | | | x | | |  | | |
| Non aderenza all'esame diagnostico | | Noncompliance of Diagnostic Test | |  | | | | | |  | |  | |  | | |  | | |  | | |  | | |  | | |  | | |  | | |  | | |  | | |  | | |  | |  |  |  |  |
| Non aderenza al regime dietetico | | | Noncompliance of Dietary Regimen | |  | |  | |  | | |  | | |  | | |  | | |  | | | |  | | |  | | |  | | |  | | |  | | |  | | |  | |  |  |  |  |  |
| Non aderenza sul volume di liquidi da assumere | | | Noncompliance of Fluid Volume | |  | |  | |  | | |  | | |  | | |  | | |  | | | |  | | |  | | |  | | |  | | |  | | |  | | |  | |  |  |  |  |  |

| **Tab 1.** *Cont.*  **Nursing Outcomes** |  | | | **References** | | | | | | | | | | | | | | | | | | | | | | | | | |  |  |  |  |  |
| --- | --- | --- | --- | --- | --- | --- | --- | --- | --- | --- | --- | --- | --- | --- | --- | --- | --- | --- | --- | --- | --- | --- | --- | --- | --- | --- | --- | --- | --- | --- | --- | --- | --- | --- |
|  |  | | | **Adamson, 2013** | | **Broekema, 2019** | | **Dening, 2016** | | **Duncan, 2021** | | **Falavigna, 2020** | | **Holdoway, 2019** | | **Husband J, 2008** | | **Jin, 2020** | | **Murray, 2017** | | **Ogston-Tuck, 2018** | | **Kelly, 2019** | **Kent, 2011** | **Shafie, 2018** | | **Simonetti, 2021** | | | | **Wood-Baker, 2012** | |  |
| Italian wording | English wording | | |  | |  | |  | |  | |  | |  | |  | |  | |  | |  | |  |  |  | |  | | | |  | |  |
| Non aderenza al regime dietetico | | | Noncompliance of Dietary Regimen |  | |  | |  | |  | |  | |  | |  | |  | |  | |  | |  |  |  | |  | | |  |  |  |  |
| Non aderenza sul volume di liquidi da assumere | | | Noncompliance of Fluid Volume |  | |  | |  | |  | |  | |  | |  | |  | |  | |  | |  |  |  | |  | | |  |  |  |  |
| Non aderenza al regime farmacologico | | | Noncompliance of Medication Regimen |  | |  | |  | |  | |  | |  | |  | |  | |  | |  | |  |  |  | |  | | |  |  |  |  |
| Non aderenza alle precauzioni di sicurezza | | Noncompliance of Safety Precautions | | |  | |  | |  | |  | |  | |  | |  | |  | |  | |  | |  | |  | | x | | | |  | |
| Non aderenza al regime terapeutico | | Noncompliance of Therapeutic Regimen | | |  | |  | |  | |  | |  | |  | |  | |  | |  | |  | |  | |  | |  | | | |  | |

| **Tab 1.** *Cont.*  **Nursing Outcomes** | |  | | **References** | | | | | | | | | | | | | | | | | | | | | | | | | | |  |  |  |  |
| --- | --- | --- | --- | --- | --- | --- | --- | --- | --- | --- | --- | --- | --- | --- | --- | --- | --- | --- | --- | --- | --- | --- | --- | --- | --- | --- | --- | --- | --- | --- | --- | --- | --- | --- |
|  | |  | | **Adamson, 2013** | **Broekema, 2019** | | **Dening, 2016** | | **Duncan, 2021** | | **Falavigna, 2020** | | **Holdoway, 2019** | | **Husband J, 2008** | | **Jin, 2020** | | **Murray, 2017** | | **Ogston-Tuck, 2018** | | **Kelly, 2019** | | **Kent, 2011** | | **Shafie, 2018** | | **Simonetti, 2021** | | | **Wood-Baker, 2012** | |  |
| Italian wording | | English wording | |  |  | |  | |  | |  | |  | |  | |  | |  | |  | |  | |  | |  | |  | | |  | |  |
| Rischi correlati al trattamento farmacologico | | Medication Risk | |  |  | |  | |  | |  | |  | |  | |  | |  | |  | |  | |  | |  | |  | | |  | |  |
| Polifarmacoterapia | | Polypharmacy | |  |  | |  | |  | |  | |  | |  | |  | |  | |  | |  | |  | | x | |  | | |  | |  |
| Alterazione endocrina | | Endocrine Alteration | |  |  | |  | |  | |  | |  | |  | |  | |  | |  | |  | |  | |  | |  | | |  | |  |
| Alterazione immunologica | | Immunologic Alteration | |  |  | |  | |  | |  | |  | |  | |  | |  | |  | |  | |  | |  | |  | | |  | |  |
| Alterazione nutrizionale | Nutrition Alteration | |  | | |  | |  | |  | |  | |  | |  | |  | |  | |  | |  | |  | |  | |  | | |  | |
| Carenza nutrizionale | Body Nutrition Deficit | |  | | |  | |  | |  | |  | |  | |  | |  | |  | |  | |  | |  | |  | |  | | |  | |

| **Tab 1.** *Cont.*  **Nursing Outcomes** | |  | | | | | **References** | | | | | | | | | | | | | | | | | | | | | | | | | | | | | | | | | | | | | | | | | | | | | | | | | | | | | |  |  |  |  |  |  |  |
| --- | --- | --- | --- | --- | --- | --- | --- | --- | --- | --- | --- | --- | --- | --- | --- | --- | --- | --- | --- | --- | --- | --- | --- | --- | --- | --- | --- | --- | --- | --- | --- | --- | --- | --- | --- | --- | --- | --- | --- | --- | --- | --- | --- | --- | --- | --- | --- | --- | --- | --- | --- | --- | --- | --- | --- | --- | --- | --- | --- | --- | --- | --- | --- | --- | --- | --- | --- |
|  | |  | | | | | **Adamson, 2013** | | **Broekema, 2019** | | | **Dening, 2016** | | | | **Duncan, 2021** | | | | **Falavigna, 2020** | | | | **Holdoway, 2019** | | | | **Husband J, 2008** | | | | | **Jin, 2020** | | | | **Murray, 2017** | | | | **Ogston-Tuck, 2018** | | | | **Kelly, 2019** | | | | **Kent, 2011** | | | | **Shafie, 2018** | | | | **Simonetti, 2021** | | | | | **Wood-Baker, 2012** | | | | |  |
| Italian wording | | English wording | | | | |  | |  | | |  | | | |  | | | |  | | | |  | | | |  | | | | |  | | | |  | | | |  | | | |  | | | |  | | | |  | | | |  | | | | |  | | | | |  |
| Rischio di carenza nutrizionale | Body Nutrition Deficit Risk | | | |  | | | | | |  | | | |  | | | |  | | | |  | | | |  | | | |  | | | |  | | | |  | | | |  | | | |  | | | |  | | | |  | | |  | | | | | |  |  |  |  |
| Eccesso nutrizionale | | | | Body Nutrition Excess | | | |  | |  | | |  | | | | |  | | | |  | | | |  | | | |  | | | |  | | | |  | | | |  | | | |  | | | |  | | | |  | | | | |  | | | | | |  | | |
| Rischio di eccesso nutrizionale | | | | Body Nutrition Excess Risk | | | |  | |  | | |  | | | | |  | | | |  | | | |  | | | |  | | | |  | | | |  | | | |  | | | |  | | | |  | | | |  | | | | |  | | | |  |  |  |  |  |
| Compromissione della deglutizione | | | | Swallowing Impairment | | | |  | |  | | |  | | | | |  | | | |  | | | |  | | | |  | | | |  | | | |  | | | |  | | | |  | | | |  | | | |  | | | | |  | | | | | |  | | |
| Compromissione del modello di alimentazione del bambino | | | Infant Feeding Pattern Impairment | | |  | | | | | | | |  | | |  | | | |  | | | |  | | | |  | | |  | | | |  | | | |  | | | |  | | | |  | | | |  | | | |  | | | |  | | |  |  |  |  |  |
| Compromissione dell'allattamento al seno | | | Breastfeeding Impairment | | |  | | | | | | | |  | | |  | | | |  | | | |  | | | |  | | |  | | | |  | | | |  | | | |  | | | |  | | | |  | | | |  | | | |  | | | | | |  | |
| Alterata regolazione dell'omeostasi corporea | | | Physical Regulation Alteration | | |  | | | | | | | |  | | |  | | | |  | | | |  | | | |  | | |  | | | |  | | | |  | | | |  | | | |  | | | |  | | | |  | | | |  | | |  |  |  |  |  |

| **Tab 1.** *Cont.*  **Nursing Outcomes** | |  | | **References** | | | | | | | | | | | | | | |  |
| --- | --- | --- | --- | --- | --- | --- | --- | --- | --- | --- | --- | --- | --- | --- | --- | --- | --- | --- | --- |
|  |  | | **Adamson, 2013** | | **Broekema, 2019** | **Dening, 2016** | **Duncan, 2021** | **Falavigna, 2020** | **Holdoway, 2019** | **Husband J, 2008** | **Jin, 2020** | **Murray, 2017** | **Ogston-Tuck, 2018** | **Kelly, 2019** | **Kent, 2011** | **Shafie, 2018** | **Simonetti, 2021** | **Wood-Baker, 2012** | |
| Italian wording | English wording | |  | |  |  |  |  |  |  |  |  |  |  |  |  |  |  | |
| Disreflessia autonomica | Autonomic Dysreflexia | |  | |  |  |  |  |  |  |  |  |  |  |  |  |  |  | |
| Ipertermia | Hyperthermia | |  | |  |  |  |  |  |  |  |  |  |  |  |  |  |  | |
| Ipotermia | Hypothermia | |  | |  |  |  |  |  |  |  |  |  |  |  |  |  |  | |
| Compromissione della termoregolazione | Thermoregulation Impairment | |  | |  |  |  |  |  |  |  |  |  |  |  |  |  |  | |
| Rischio di infezione | Infection Risk | |  | |  |  |  |  |  |  |  |  |  |  |  |  |  |  | |
| Compromissione della capacità adattiva intracranica | Intracranial Adaptive Capacity Impairment | | | |  |  |  |  |  |  |  |  |  |  |  |  |  |  | |

| **Tab 1.** *Cont.*  **Nursing Outcomes** |  | | **References** | | | | | | | | | | | | | | | | | | | | | | | | | | |  |  |  |  |  |
| --- | --- | --- | --- | --- | --- | --- | --- | --- | --- | --- | --- | --- | --- | --- | --- | --- | --- | --- | --- | --- | --- | --- | --- | --- | --- | --- | --- | --- | --- | --- | --- | --- | --- | --- |
|  |  | | **Adamson, 2013** | | **Broekema, 2019** | | **Dening, 2016** | | **Duncan, 2021** | | **Falavigna, 2020** | | **Holdoway, 2019** | | **Husband J, 2008** | | **Jin, 2020** | | **Murray, 2017** | | **Ogston-Tuck, 2018** | | **Kelly, 2019** | | **Kent, 2011** | | **Shafie, 2018** | | **Simonetti, 2021** | | | **Wood-Baker, 2012** |  |  |
| Italian wording | English wording | |  | |  | |  | |  | |  | |  | |  | |  | |  | |  | |  | |  | |  | |  | | |  |  |  |
| Alterazione della respirazione | Respiration Alteration |  | |  | |  | |  | |  | |  | |  | |  | |  | |  | |  | |  | |  | |  | | |  | | | |
| Compromissione della liberazione delle vie aeree | Airway Clearance Impairment |  | |  | |  | |  | |  | |  | |  | |  | |  | |  | |  | |  | |  | |  | |  |  |  |  |  |
| Compromissione del modello di respirazione | Breathing Pattern Impairment |  | |  | |  | |  | |  | |  | |  | |  | |  | |  | |  | |  | |  | |  | |  |  |  |  |  |
| Compromissione degli scambi gassosi | Gas Exchange Impairment |  | |  | |  | |  | |  | |  | |  | |  | |  | |  | |  | |  | |  | |  | |  | | | |  |

| **Tab 1.** *Cont.*  **Nursing Outcomes** | |  | | **References** | | | | | | | | | | | | | | | | | | | | | | | | | | | | | |
| --- | --- | --- | --- | --- | --- | --- | --- | --- | --- | --- | --- | --- | --- | --- | --- | --- | --- | --- | --- | --- | --- | --- | --- | --- | --- | --- | --- | --- | --- | --- | --- | --- | --- |
|  | |  | | **Adamson, 2013** | | **Broekema, 2019** | | **Dening, 2016** | | **Duncan, 2021** | | **Falavigna, 2020** | | **Holdoway, 2019** | | **Husband J, 2008** | | **Jin, 2020** | | **Murray, 2017** | | **Ogston-Tuck, 2018** | | **Kelly, 2019** | | **Kent, 2011** | | **Shafie, 2018** | | **Simonetti, 2021** | | | **Wood-Baker, 2012** |
| Italian wording | | English wording | |  | |  | |  | |  | |  | |  | |  | |  | |  | |  | |  | |  | |  | |  | | |  |
| Compromissione dello svezzamento dal ventilatore | | Ventilatory Weaning Impairment | | | |  | |  | |  | |  | |  | |  | |  | |  | |  | |  | |  | |  | |  | | |  |
| Alterazione della capacità di svolgere un ruolo | | Role Performance Alteration | | | |  | |  | |  | |  | |  | |  | |  | |  | |  | |  | |  | |  | |  | | |  |
| Conflitto nel ruolo genitoriale | Parental Role Conflict | |  | |  | |  | |  | |  | |  | |  | |  | |  | |  | |  | |  | |  | |  | |  |  |  |
| Alterazione delle capacità di accudimento | Parenting Alteration | |  | |  | |  | |  | |  | |  | |  | |  | |  | |  | |  | | x | |  | |  | |  |  |  |
| Disfunzione sessuale | Sexual Dysfunction | |  | |  | |  | |  | |  | |  | |  | |  | |  | |  | |  | |  | |  | |  | |  |  |  |
| Tensione nel ruolo di caregiver | Caregiver Role Strain | | x | |  | |  | |  | |  | |  | |  | |  | |  | |  | |  | |  | |  | |  | |  |  |  |

| **Tab 1.** *Cont.*  **Nursing Outcomes** |  | **References** | | | | | | | | | | | | | |  |  |
| --- | --- | --- | --- | --- | --- | --- | --- | --- | --- | --- | --- | --- | --- | --- | --- | --- | --- |
|  |  | **Adamson, 2013** | **Broekema, 2019** | **Dening, 2016** | **Duncan, 2021** | **Falavigna, 2020** | **Holdoway, 2019** | **Husband J, 2008** | **Jin, 2020** | **Murray, 2017** | **Ogston-Tuck, 2018** | **Kelly, 2019** | **Kent, 2011** | **Shafie, 2018** | **Simonetti, 2021** | | **Wood-Baker, 2012** |
| Italian wording | English wording |  |  |  |  |  |  |  |  |  |  |  |  |  |  | |  |
| Compromissione della comunicazione | Communication Impairment | |  |  |  |  |  |  |  |  |  |  |  |  |  | |  |
| Compromissione della comunicazione verbale | Verbal Impairment |  |  |  |  |  |  |  |  |  |  |  |  |  |  | |  |
| Alterazione dei processi familiari | Family Processes Alteration | x | x |  |  |  |  |  |  |  |  |  |  |  |  | |  |
| Cambiamento o variazione del normale funzionamento del gruppo familiare | Sexuality Patterns Alteration | |  |  |  |  |  |  |  |  |  |  |  |  |  | |  |
| Alterazione della socializzazione | Socialization Alteration |  |  |  |  |  |  |  |  |  |  |  |  |  |  | |  |

| **Tab 1.** *Cont.*  **Nursing Outcomes** |  | **References** | | | | | | | | | | | | | |  |  |
| --- | --- | --- | --- | --- | --- | --- | --- | --- | --- | --- | --- | --- | --- | --- | --- | --- | --- |
|  |  | **Adamson, 2013** | **Broekema, 2019** | **Dening, 2016** | **Duncan, 2021** | **Falavigna, 2020** | **Holdoway, 2019** | **Husband J, 2008** | **Jin, 2020** | **Murray, 2017** | **Ogston-Tuck, 2018** | **Kelly, 2019** | **Kent, 2011** | **Shafie, 2018** | **Simonetti, 2021** | | **Wood-Baker, 2012** |
| Italian wording | English wording |  |  |  |  |  |  |  |  |  |  |  |  |  |  | |  |
| Alterazione delle interazioni sociali | Social Interaction Alteration | |  |  |  |  |  |  |  |  |  |  |  |  |  | |  |
| Isolamento sociale | Social Isolation |  |  |  |  |  |  |  |  |  |  |  |  |  |  | |  |
| Sindrome da stress da trasferimento | Relocation Stress Syndrome | |  |  |  |  |  |  |  |  |  |  |  |  |  | |  |
| Italian wording | English wording |  |  |  |  |  |  |  |  |  |  |  |  |  |  | |  |
| Rischio di lesione | Injury Risk |  |  |  |  |  |  |  |  |  |  |  |  |  |  | |  |
| Rischio di inalazione | Aspiration Risk |  |  |  |  |  |  |  |  |  |  |  |  |  |  | |  |
| Sindrome da immobilizzazione | Disuse Syndrome |  |  |  |  |  |  |  |  |  |  |  |  |  |  | |  |
| Rischio di avvelenamento | Poisoning Risk |  |  |  |  |  |  |  |  |  |  |  |  |  |  | |  |
| Rischio di asfissia | Suffocation Risk |  |  |  |  |  |  |  | x |  |  |  |  |  |  | |  |
| **Tab 1.** *Cont.*  **Nursing Outcomes** |  | **References** | | | | | | | | | | | | | |  |  |
|  |  | **Adamson, 2013** | **Broekema, 2019** | **Dening, 2016** | **Duncan, 2021** | **Falavigna, 2020** | **Holdoway, 2019** | **Husband J, 2008** | **Jin, 2020** | **Murray, 2017** | **Ogston-Tuck, 2018** | **Kelly, 2019** | **Kent, 2011** | **Shafie, 2018** | **Simonetti, 2021** | | **Wood-Baker, 2012** |
| Rischio di lesione traumatica | Trauma Risk |  |  |  |  |  |  |  |  |  |  |  |  |  |  | |  |
| Rischio di caduta | Fall Risk |  |  |  |  |  |  |  |  |  |  |  |  |  |  | |  |
| Rischio di comportamento violento | Violence Risk |  |  |  |  |  |  |  |  |  |  |  |  | x |  | |  |
| Rischio di suicidio | Suicide Risk |  |  |  |  |  |  |  |  |  |  |  |  |  |  | |  |
| Rischio di autolesionismo | Self mutilation Risk |  |  |  |  |  |  |  |  |  |  |  |  |  |  | |  |
| Rischio di lesione perioperatoria | Perioperative Injury Risk |  |  |  |  |  |  |  |  |  |  |  |  |  |  | |  |
| Lesione da posizionamento perioperatorio | Perioperative Positioning Injury | |  |  |  |  |  |  |  |  |  |  |  |  |  | |  |
| Ritardo nella guarigione postchirurgica | Surgical Recovery Delay |  |  |  |  |  |  |  |  |  |  |  |  |  |  | |  |
| Abuso di sostanze | Substance Abuse |  |  |  |  |  |  |  |  |  |  |  |  |  |  | |  |

| **Tab 1.** *Cont.*  **Nursing Outcomes** |  | **References** | | | | | | | | | | | | | |  |  |
| --- | --- | --- | --- | --- | --- | --- | --- | --- | --- | --- | --- | --- | --- | --- | --- | --- | --- |
|  |  | **Adamson, 2013** | **Broekema, 2019** | **Dening, 2016** | **Duncan, 2021** | **Falavigna, 2020** | **Holdoway, 2019** | **Husband J, 2008** | **Jin, 2020** | **Murray, 2017** | **Ogston-Tuck, 2018** | **Kelly, 2019** | **Kent, 2011** | **Shafie, 2018** | **Simonetti, 2021** | | **Wood-Baker, 2012** |
| Italian wording | English wording |  |  |  |  |  |  |  |  |  |  |  |  |  |  | |  |
| Abuso di tabacco | Tobacco Abuse |  |  |  |  |  |  |  |  |  |  |  |  |  |  | |  |
| Abuso di alcol | Alcohol Abuse |  |  |  |  |  |  |  |  |  |  |  |  |  |  | |  |
| Abuso di farmaci | Drug Abuse |  |  |  |  |  |  |  |  |  |  |  |  |  |  | |  |
| Deficit nella capacità di lavarsi | Bathing/Hygiene Deficit |  |  |  |  |  |  |  |  | x |  |  |  |  |  | |  |
| Deficit nel vestirsi/curare il proprio aspetto | Dressing/Grooming Deficit |  |  |  |  |  |  |  |  |  |  |  |  |  |  | |  |
| Deficit nella capacità di alimentarsi | Feeding Deficit |  |  |  |  |  |  |  |  |  |  |  |  |  |  | |  |
| Deficit di self-care | Self Care Deficit |  |  |  |  |  |  |  |  |  |  |  |  |  |  | |  |

| **Tab 1.** *Cont.*  **Nursing Outcomes** |  | | **References** | | | | | | | | | | | | | | | | | | | | | | | | | | |  |  |  |
| --- | --- | --- | --- | --- | --- | --- | --- | --- | --- | --- | --- | --- | --- | --- | --- | --- | --- | --- | --- | --- | --- | --- | --- | --- | --- | --- | --- | --- | --- | --- | --- | --- |
|  |  | | **Adamson, 2013** | | **Broekema, 2019** | | **Dening, 2016** | | **Duncan, 2021** | | **Falavigna, 2020** | | **Holdoway, 2019** | | **Husband J, 2008** | | **Jin, 2020** | | **Murray, 2017** | | **Ogston-Tuck, 2018** | | **Kelly, 2019** | | **Kent, 2011** | | **Shafie, 2018** | | **Simonetti, 2021** | | | **Wood-Baker, 2012** |
| Italian wording | English wording | |  | |  | |  | |  | |  | |  | |  | |  | |  | |  | |  | |  | |  | |  | | |  |
| Alterazione delle attività di vita quotidiana (ADL) | Activities of Daily Living (ADLs) Alteration |  | |  | |  | |  | |  | |  | |  | |  | |  | |  | |  | |  | |  | |  | |  |  |  |
| Alterazione delle attività strumentali di vita quotidiana (IADL) | Instrumental Activities of Daily Living (IADLs) Alteration |  | |  | |  | |  | |  | |  | |  | |  | |  | |  | |  | |  | |  | | | | |  |  |
| Deficit nella capacità di utilizzare il gabinetto | Toileting Deficit | |  | |  | |  | |  | |  | |  | |  | |  | |  | |  | |  | |  | |  | |  | | |  |
| Ansia | Anxiety | |  | |  | |  | |  | |  | |  | |  | |  | | x | |  | |  | |  | |  | |  | | |  |
| Paura | Fear | |  | |  | |  | |  | |  | |  | |  | |  | | x | |  | |  | |  | |  | |  | | |  |

| **Tab 1.** *Cont.*  **Nursing Outcomes** |  | | **References** | | | | | | | | | | | | | | | | | | | | | | | | | | |  |  |  |  |
| --- | --- | --- | --- | --- | --- | --- | --- | --- | --- | --- | --- | --- | --- | --- | --- | --- | --- | --- | --- | --- | --- | --- | --- | --- | --- | --- | --- | --- | --- | --- | --- | --- | --- |
|  |  | | **Adamson, 2013** | | **Broekema, 2019** | | **Dening, 2016** | | **Duncan, 2021** | | **Falavigna, 2020** | | **Holdoway, 2019** | | **Husband J, 2008** | | **Jin, 2020** | | **Murray, 2017** | | **Ogston-Tuck, 2018** | | **Kelly, 2019** | | **Kent, 2011** | | **Shafie, 2018** | | **Simonetti, 2021** | | | **Wood-Baker, 2012** |  |
| Italian wording | English wording | |  | |  | |  | |  | |  | |  | |  | |  | |  | |  | |  | |  | |  | |  | | |  |  |
| Alterazione dell'attribuzione di senso e significato | Meaningfulness Alteration |  | |  | |  | |  | |  | |  | |  | |  | |  | |  | |  | |  | |  | |  | | |  | | |
| Mancanza di speranza | Hopelessness |  | |  | |  | |  | |  | |  | |  | |  | |  | |  | |  | |  | |  | |  | | |  | | |
| Senso di impotenza | Powerlessness |  | |  | |  | |  | |  | |  | |  | |  | |  | |  | |  | |  | |  | |  | | |  | | |
| Alterazione del concetto di sé | Self Concept Alteration | |  | |  | |  | |  | |  | |  | |  | |  | |  | |  | |  | |  | |  | |  | | |  |  |
| Disturbo dell'immagine corporea | Body Image Disturbance | |  | |  | |  | |  | |  | |  | |  | |  | |  | |  | |  | |  | |  | |  | | |  |  |
| Disturbo dell'identità personale | Personal Identity Disturbance | | | |  | |  | |  | |  | |  | |  | |  | |  | |  | |  | |  | |  | |  | | |  |  |

| **Tab 1.** *Cont.*  **Nursing Outcomes** |  | | **References** | | | | | | | | | | | | | | | | | | | | | | | | | | |  |  |  |  |
| --- | --- | --- | --- | --- | --- | --- | --- | --- | --- | --- | --- | --- | --- | --- | --- | --- | --- | --- | --- | --- | --- | --- | --- | --- | --- | --- | --- | --- | --- | --- | --- | --- | --- |
|  |  | | **Adamson, 2013** | | **Broekema, 2019** | | **Dening, 2016** | | **Duncan, 2021** | | **Falavigna, 2020** | | **Holdoway, 2019** | | **Husband J, 2008** | | **Jin, 2020** | | **Murray, 2017** | | **Ogston-Tuck, 2018** | | **Kelly, 2019** | | **Kent, 2011** | | **Shafie, 2018** | | **Simonetti, 2021** | | | **Wood-Baker, 2012** |  |
| Italian wording | English wording | |  | |  | |  | |  | |  | |  | |  | |  | |  | |  | |  | |  | |  | |  | | |  |  |
| Disturbo cronico di bassa autostima | Chronic Low Self-Esteem Disturbance |  | |  | |  | |  | |  | |  | |  | |  | |  | |  | |  | |  | |  | |  | |  |  |  |  |
| Disturbo situazionale di autostima | Situational Self-Esteem Disturbance |  | |  | |  | |  | |  | |  | |  | |  | |  | |  | |  | |  | |  | |  | |  |  |  |  |
| Alterazione della percezione sensoriale | Sensory Perceptual Alteration |  | |  | |  | |  | |  | |  | |  | |  | |  | |  | |  | |  | |  | |  | |  |  |  |  |
| Alterazione dell'udito | Auditory Alteration |  | |  | |  | |  | |  | |  | |  | |  | |  | |  | |  | |  | |  | |  | | |  | | |
| Alterazione del gusto | Gustatory Alteration |  | |  | |  | |  | |  | |  | |  | |  | |  | |  | |  | |  | |  | |  | | |  | | |
| Alterazione cinestetica | Kinesthetic Alteration |  | |  | |  | |  | |  | |  | |  | |  | |  | |  | |  | |  | |  | |  | | |  | | |

| **Tab 1.** *Cont.*  **Nursing Outcomes** |  | | **References** | | | | | | | | | | | | | | | | | | | | | | | | | | |  |  |  |  |
| --- | --- | --- | --- | --- | --- | --- | --- | --- | --- | --- | --- | --- | --- | --- | --- | --- | --- | --- | --- | --- | --- | --- | --- | --- | --- | --- | --- | --- | --- | --- | --- | --- | --- |
|  |  | | **Adamson, 2013** | | **Broekema, 2019** | | **Dening, 2016** | | **Duncan, 2021** | | **Falavigna, 2020** | | **Holdoway, 2019** | | **Husband J, 2008** | | **Jin, 2020** | | **Murray, 2017** | | **Ogston-Tuck, 2018** | | **Kelly, 2019** | | **Kent, 2011** | | **Shafie, 2018** | | **Simonetti, 2021** | | | **Wood-Baker, 2012** |  |
| Italian wording | English wording | |  | |  | |  | |  | |  | |  | |  | |  | |  | |  | |  | |  | |  | |  | | |  |  |
| Alterazione dell'olfatto | Olfactory Alteration |  | |  | |  | |  | |  | |  | |  | |  | |  | |  | |  | |  | |  | |  | | |  | | |
| Alterazione del tatto | Tactile Alteration |  | |  | |  | |  | |  | |  | |  | |  | |  | |  | |  | |  | |  | |  | | |  | | |
| Eminegligenza | Unilateral Neglect |  | |  | |  | |  | |  | |  | |  | |  | |  | |  | |  | |  | |  | |  | | |  | | |
| Alterazione della vista | Visual Alteration |  | |  | |  | |  | |  | |  | |  | |  | |  | |  | |  | |  | |  | |  | | |  | | |
| Alterazione del comfort | Comfort Alteration |  | |  | |  | |  | |  | |  | |  | |  | |  | |  | |  | |  | |  | |  | | |  | | |
| Dolore | Pain |  | |  | |  | |  | |  | |  | |  | |  | |  | |  | |  | |  | |  | |  | | |  | | |
| Dolore acuto | Acute Pain |  | |  | |  | |  | |  | |  | |  | |  | |  | |  | |  | |  | |  | |  | | |  | | |

| **Tab 1.** *Cont.*  **Nursing Outcomes** |  | | **References** | | | | | | | | | | | | | | | | | | | | | | | | | | |  |  |  |  |
| --- | --- | --- | --- | --- | --- | --- | --- | --- | --- | --- | --- | --- | --- | --- | --- | --- | --- | --- | --- | --- | --- | --- | --- | --- | --- | --- | --- | --- | --- | --- | --- | --- | --- |
|  |  | | **Adamson, 2013** | | **Broekema, 2019** | | **Dening, 2016** | | **Duncan, 2021** | | **Falavigna, 2020** | | **Holdoway, 2019** | | **Husband J, 2008** | | **Jin, 2020** | | **Murray, 2017** | | **Ogston-Tuck, 2018** | | **Kelly, 2019** | | **Kent, 2011** | | **Shafie, 2018** | | **Simonetti, 2021** | | | **Wood-Baker, 2012** |  |
| Italian wording | English wording | |  | |  | |  | |  | |  | |  | |  | |  | |  | |  | |  | |  | |  | |  | | |  |  |
| Dolore cronico | Chronic Pain |  | |  | |  | |  | |  | |  | |  | |  | |  | |  | |  | |  | |  | |  | | |  | | |
| Alterazione dell'integrità cutanea | Skin Integrity Alteration |  | |  | |  | |  | |  | |  | |  | |  | |  | |  | |  | |  | |  | |  | | |  | | |
| Compromissione della mucosa orale | Oral Mucous Membranes Impairment |  | |  | |  | |  | |  | |  | |  | |  | |  | |  | |  | |  | |  | |  | |  |  |  |  |
| Compromissione dell'integrità cutanea | Skin Integrity Impairment |  | |  | |  | |  | |  | |  | |  | |  | |  | |  | |  | |  | |  | |  | | |  | | |

| **Tab 1.** *Cont.*  **Nursing Outcomes** |  | | **References** | | | | | | | | | | | | | | | | | | | | | | | | | | |  |  |  |  |
| --- | --- | --- | --- | --- | --- | --- | --- | --- | --- | --- | --- | --- | --- | --- | --- | --- | --- | --- | --- | --- | --- | --- | --- | --- | --- | --- | --- | --- | --- | --- | --- | --- | --- |
|  |  | | **Adamson, 2013** | | **Broekema, 2019** | | **Dening, 2016** | | **Duncan, 2021** | | **Falavigna, 2020** | | **Holdoway, 2019** | | **Husband J, 2008** | | **Jin, 2020** | | **Murray, 2017** | | **Ogston-Tuck, 2018** | | **Kelly, 2019** | | **Kent, 2011** | | **Shafie, 2018** | | **Simonetti, 2021** | | | **Wood-Baker, 2012** |  |
| Italian wording | English wording | |  | |  | |  | |  | |  | |  | |  | |  | |  | |  | |  | |  | |  | |  | | |  |  |
| Rischio di compromissione dell'integrità cutanea | Skin Integrity Impairment Risk |  | |  | |  | |  | |  | |  | |  | |  | |  | |  | |  | |  | |  | |  | |  |  |  |  |
| Ferita chirurgica | Skin Incision |  | |  | |  | |  | |  | |  | |  | |  | |  | |  | |  | |  | |  | |  | | |  | | |
| Reazione allergica al lattice | Latex Allergy Response |  | |  | |  | |  | |  | |  | |  | |  | |  | |  | |  | |  | |  | |  | | |  | | |
| Alterazione neurovascolare periferica | Peripheral Alteration |  | |  | |  | |  | |  | |  | |  | |  | |  | |  | |  | |  | |  | |  | | |  | | |
| Alterazione della perfusione tessutale | Tissue Perfusion Alteration |  | |  | |  | |  | |  | |  | |  | |  | |  | |  | |  | |  | |  | |  | |  |  |  |  |
| Alterazione dell'eliminazione urinaria | Urinary Elimination Alteration |  | |  | |  | |  | |  | |  | |  | |  | |  | |  | |  | |  | |  | |  | |  |  |  |  |

| **Tab 1.** *Cont.*  **Nursing Outcomes** |  | | **References** | | | | | | | | | | | | | | | | | | | | | | | | | | |  |  |  |  |
| --- | --- | --- | --- | --- | --- | --- | --- | --- | --- | --- | --- | --- | --- | --- | --- | --- | --- | --- | --- | --- | --- | --- | --- | --- | --- | --- | --- | --- | --- | --- | --- | --- | --- |
|  |  | | **Adamson, 2013** | | **Broekema, 2019** | | **Dening, 2016** | | **Duncan, 2021** | | **Falavigna, 2020** | | **Holdoway, 2019** | | **Husband J, 2008** | | **Jin, 2020** | | **Murray, 2017** | | **Ogston-Tuck, 2018** | | **Kelly, 2019** | | **Kent, 2011** | | **Shafie, 2018** | | **Simonetti, 2021** | | | **Wood-Baker, 2012** |  |
| Italian wording | English wording | |  | |  | |  | |  | |  | |  | |  | |  | |  | |  | |  | |  | |  | |  | | |  |  |
| Incontinenza urinaria funzionale | Functional Urinary Incontinence |  | |  | |  | |  | |  | |  | |  | |  | |  | |  | |  | |  | |  | |  | |  |  |  |  |
| Incontinenza urinaria riflessa | Reflex Urinary Incontinence |  | |  | |  | |  | |  | |  | |  | |  | |  | |  | |  | |  | |  | |  | |  |  |  |  |
| Incontinenza urinaria da stress | Stress Urinary Incontinence |  | |  | |  | |  | |  | |  | |  | |  | |  | |  | |  | |  | |  | |  | |  |  |  |  |
| Incontinenza urinaria da urgenza | Urge Urinary Incontinence |  | |  | |  | |  | |  | |  | |  | |  | |  | |  | |  | |  | |  | |  | | |  | | |
| Ritenzione urinaria | Urinary Retention |  | |  | |  | |  | |  | |  | |  | |  | |  | |  | |  | |  | |  | |  | | |  | | |

| **Tab 1.** *Cont.*  **Nursing Outcomes** |  | | **References** | | | | | | | | | | | | | | | | | | | | | | | | | | | | | | | |  |  |  |  |  |  |
| --- | --- | --- | --- | --- | --- | --- | --- | --- | --- | --- | --- | --- | --- | --- | --- | --- | --- | --- | --- | --- | --- | --- | --- | --- | --- | --- | --- | --- | --- | --- | --- | --- | --- | --- | --- | --- | --- | --- | --- | --- |
|  |  | | **Adamson, 2013** | | **Broekema, 2019** | | **Dening, 2016** | | **Duncan, 2021** | | **Falavigna, 2020** | | **Holdoway, 2019** | | **Husband J, 2008** | | **Jin, 2020** | | **Murray, 2017** | | | **Ogston-Tuck, 2018** | | | **Kelly, 2019** | | | **Kent, 2011** | | | **Shafie, 2018** | | | **Simonetti, 2021** | | | | **Wood-Baker, 2012** |  |  |
| Italian wording | English wording | |  | |  | |  | |  | |  | |  | |  | |  | |  | | |  | | |  | | |  | | |  | | |  | | | |  |  |  |
| Alterazione della funzionalità renale | Renal Alteration |  | |  | |  | |  | |  | |  | |  | |  | |  | | |  | | |  | | |  | | |  | | |  | | | |  | | |  |
| Rischio associato al processo riproduttivo | Rischio associato al processo riproduttivo |  | |  | | x | |  | |  | |  | |  | |  | |  | | |  | | |  | | |  | | |  | | |  | |  |  |  |  |  |  |
| Rischio di gravidanza indesiderata | Fertility Risk |  | |  | |  | |  | |  | |  | |  | |  | |  | | |  | | |  | | |  | | |  | | |  | | | |  | | |  |
| Rischio di infertilità | Infertility Risk |  | |  | |  | |  | |  | |  | |  | |  | |  | | |  | | |  | | |  | | |  | | |  | | | |  | | |  |
| Rischio associato alla contraccezione | Contraception Risk |  | |  | |  | |  | |  | |  | |  | |  | |  | |  | | |  | | |  | | |  | | |  | | | |  | | | | |
| Rischio associato al periodo perinatale | Perinatal Risk |  | |  | |  | |  | |  | |  | |  | |  | |  | |  | | |  | | |  | | |  | | |  | | | |  | | | | |
| Rischio associato alla gravidanza | Pregnancy Risk |  | |  | |  | |  | |  | |  | |  | |  | |  | |  | | |  | | |  | | |  | | |  | | | |  | | | | |

| **Tab 1.** *Cont.*  **Nursing Outcomes** |  | | **References** | | | | | | | | | | | | | | | | | | | | | | | | | | |  |  |  |  |
| --- | --- | --- | --- | --- | --- | --- | --- | --- | --- | --- | --- | --- | --- | --- | --- | --- | --- | --- | --- | --- | --- | --- | --- | --- | --- | --- | --- | --- | --- | --- | --- | --- | --- |
|  |  | | **Adamson, 2013** | | **Broekema, 2019** | | **Dening, 2016** | | **Duncan, 2021** | | **Falavigna, 2020** | | **Holdoway, 2019** | | **Husband J, 2008** | | **Jin, 2020** | | **Murray, 2017** | | **Ogston-Tuck, 2018** | | **Kelly, 2019** | | **Kent, 2011** | | **Shafie, 2018** | | **Simonetti, 2021** | | | **Wood-Baker, 2012** |  |
| Italian wording | English wording | |  | |  | |  | |  | |  | |  | |  | |  | |  | |  | |  | |  | |  | |  | | |  |  |
| Rischio associato al travaglio | Labor Risk |  | |  | |  | |  | |  | |  | |  | |  | |  | |  | |  | |  | |  | |  | | |  | | |
| Rischio associato al parto | Delivery Risk |  | |  | |  | |  | |  | |  | |  | |  | |  | |  | |  | |  | |  | |  | | |  | | |
| Rischio associato al post partum | Postpartum Risk |  | |  | |  | |  | |  | |  | |  | |  | |  | |  | |  | |  | |  | |  | | |  | | |
| Alterazione della crescita e dello sviluppo | Growth and Development Alteration |  | |  | |  | |  | |  | |  | |  | |  | |  | |  | |  | |  | |  | |  | |  |  |  |  |
